# Intravenous patient-controlled analgesia plus psychoeducational intervention for acute postoperative pain in patients with pulmonary nodules after thoracoscopic surgery: a retrospective cohort study

**DOI:** 10.1186/s12871-021-01505-4

**Published:** 2021-11-13

**Authors:** Sha Li, Xian Ding, Yong Zhao, Xiao Chen, Jianfeng Huang

**Affiliations:** 1 Department of Anesthesiology, Affiliated Hospital of Jiangnan University, 1000 Hefeng Road, Wuxi, 214125 Jiangsu People’s Republic of China; 2 Department of Thoracic and Cardiovascular Surgery, Affiliated Hospital of Jiangnan University, Wuxi, Jiangsu China; 3 Department of Radiation Oncology, Affiliated Hospital of Jiangnan University, 1000 Hefeng Road, Wuxi, 214125 Jiangsu People’s Republic of China

**Keywords:** Pulmonary nodule, Thoracoscopic surgery, Acute postoperative pain, Intravenous patient-controlled analgesia, Psychoeducational intervention

## Abstract

**Background:**

The association of psychological factors with postoperative pain has been well documented. The incorporation of psychoeducational intervention into a standard analgesia protocol seems to be an attractive approach for the management of acute postoperative pain. Our study aimed to evaluate the impact of psychoeducational intervention on acute postoperative pain in pulmonary nodule (PN) patients treated with thoracoscopic surgery.

**Methods:**

In this study, 76 PN patients treated with thoracoscopic surgery and intravenous patient-controlled analgesia (IV-PCA) plus psychoeducational evaluation and intervention were selected as the psychoeducational intervention group (PG). Another 76 PN patients receiving IV-PCA without psychoeducational intervention after thoracoscopic surgery, treated as the control group (CG), were identified from the hospital database and matched pairwise with PG patients according to age, sex, preoperative body mass index (BMI), opioid medications used for IV-PCA and the educational attainment of patients.

**Results:**

The most common psychological disorders were anxiety and interpersonal sensitivity, which were recorded from 82.9% (63/76) and 63.2% (48/76) of PG patients. The numerical rating scale (NRS) pain scores of the PG patients were significantly lower than those of the CG patients at 2 and 24 h after surgery (*P* < 0.001). Total opioid consumption for acute postoperative pain in the PG was 52.1 mg of morphine equivalent, which was significantly lower than that (67.8 mg) in the CG (*P* = 0.038). PG patients had a significantly lower incidence of rescue analgesia than CG patients (28.9% vs. 44.7%, *P* = 0.044). Nausea/vomiting was the most common side effect of opioid medications, recorded for 3 (3.9%) PG patients and 10 (13.2%) CG patients (*P* = 0.042). In addition, no significant difference was observed between PG and CG patients in terms of grade 2 or higher postoperative complications (10.5% vs. 17.1%, *P* = 0.240).

**Conclusions:**

Psychoeducational intervention for PN patients treated with thoracoscopic surgery resulted in reduced acute postoperative pain, less opioid consumption and fewer opioid-related side effects.

## Background

Pulmonary nodules (PNs) are increasingly detected with extensive use of chest computed tomography (CT) scans [1]. The standard treatment for nodules with a high probability of malignancy is video-assisted thoracoscopic surgery (VATS), which has been shown to yield less trauma to the chest wall, less pain, faster recovery and fewer complications than the open approach [2–4]. However, VATS is also accompanied by moderate to severe postoperative pain in some patients, which is one of the most disturbing complaints after surgery [5]. A number of studies have demonstrated that adequate pain relief after surgery is essential for mitigating suffering, promoting rehabilitation and reducing complications [6, 7]. Intravenous patient-controlled analgesia (IV-PCA) with continuous infusion of opioids upon the patient’s individual analgesic needs is widely used to manage acute postoperative pain [8, 9]. However, the side effects of opioid medications, such as nausea, vomiting, pruritus, urinary retention and respiratory depression, can limit their effectiveness in some patients. Hence, the optimum strategy for postoperative analgesia remains a subject of debate.

The association of psychological factors with postoperative pain and surgical recovery has been well documented [6, 10, 11]. Surgery-related perioperative stress and negative psychological states such as anxiety, depression, and catastrophising attitudes have been demonstrated to extensively affect patients’ neuroendocrine pathways and immune function, thereby leading to more serious acute pain and impaired recovery after surgery. As a result, psychological interventions, including cognitive-behavioural treatment, relaxation, mindfulness-oriented tasks, and supportive care, aiming to decrease postoperative pain and improve the quality of clinical care have been explored and shown to be effective in breast, cardiac, abdominal, and orthopaedic surgery patients, particularly for those with maladaptive psychological features [12–14]. However, there is little research focusing on the effect of psychological interventions on surgical outcomes for PN patients after thoracoscopic surgery.

In this matched-pair study, 152 PN patients who underwent thoracoscopic surgery and IV-PCA with or without psychoeducational intervention were retrospectively analysed. The aim of this work was to evaluate the impact of psychoeducational intervention on acute postoperative pain, opioid consumption, side effects of opioid medications and postoperative complications for PN patients treated by thoracoscopic surgery.

## Methods

### Patient population

Inclusion criteria: age 18 years or older; clinical diagnosis of PN with high probability of malignancy; treatment with single-port thoracoscopic wedge resection surgery; postoperative analgesia with IV-PCA; American Society of Anaesthesiologists physical status (ASA-PS) grade 1 or 2; no pain 72 h prior to the surgery.

Exclusion criteria: incomplete medical documentation; multiport thoracoscopic surgery that might increase postoperative pain; intraoperative conversion to an open approach; presence of anxiety disorders or other significant alterations of cognitive impairment or mental status or visual and auditory deficits; history of antitumour therapy, psychopharmacological drug use, or chronic opioid use for any reason; concomitant malignant disease; considerable cardiopulmonary morbidity; hypohepatia or renal insufficiency.

Seventy-six eligible patients with PN who underwent IV-PCA plus psychoeducational intervention after thoracoscopic surgery at the Affiliated Hospital of Jiangnan University between June 2018 and November 2019 were selected as the psychoeducational intervention group (PG). All PG patients provided written informed consent prior to IV-PCA and psychoeducational intervention. Another 76 PN patients receiving IV-PCA without psychoeducational intervention, included as the control group (CG), were identified from the electronic hospital database and matched pairwise with the PG according to age, sex, preoperative body mass index (BMI), opioid medications used for IV-PCA and the educational attainment of patients. Patient characteristics at baseline in both groups are detailed in Table 1.

**Table 1:** Baseline characteristics of patients

Characteristics	PG	CG	***P***-value
	n (%)	n (%)	
**Age, years**			0.870
< 46	34(44.7)	33(43.4)	
≥ 46	42(55.3)	43(56.6)	
**Gender**			0.744
Male	43(56.6)	41(53.9)	
Female	33(43.4)	35(46.1)	
**BMI, kg/m**^**2**^			0.940
< 18.5	8(10.5)	8(10.5)	
18.5–24.9	42(55.3)	40(52.6)	
≥ 25	26(34.2)	28(36.9)	
**Opioid medications used for IV-PCA**			1.000
Hydromorphone	23(30.3)	23(30.3)	
Sufentanil	28(36.8)	28(36.8)	
Fentanyl	25(32.9)	25(32.9)	
**Educational attainment**			0.840
Senior high school or below	15(19.7)	16(21.1)	
College or above	61(80.3)	60(78.9)	

*Abbreviations*: *PG* psychoeducational intervention group, *CG* control group, *BMI* body mass index, *IV-PCA* intravenous patient-controlled analgesia

### Anaesthesia and analgesia protocol

All patients in both groups received general anaesthesia according to the following protocols. Briefly, intravenous administration of 1–3 mg of midazolam, 1.5–2.5 mg/kg propofol, 4 μg/kg fentanyl and 0.6 mg/kg rocuronium was performed for induction anaesthesia. Inhalation of sevoflurane (1.0%) and continuous infusion of remifentanil (4–18 μg/kg·h), propofol (2–5 mg/kg·h) and rocuronium (0.3 mg/kg·h) were performed for the maintenance of anaesthesia. The bispectral index (BIS) was used to guide the dose of anaesthetic for all patients. Other drugs affecting analgesia intraoperatively, such as magnesium, clonidine, paracetamol, and piritramide, were not administered to any patient.

All patients received IV-PCA for postoperative pain management. An opioid agent (1 μg/ml sufentanil, 10 μg/ml fentanyl or 100 μg/ml hydromorphone) was administered at a total volume of 100 ml. The analgesic was infused basally at a rate of 2 ml/h with a bolus dose of 5 ml and a 2 ml bolus with 15-min lockout. Most patients used the total amount of IV-PCA within the first 2 postoperative days (PODs), and additional opioid analgesics (rescue analgesia) were prescribed by the anaesthesiologists or surgeons upon patient request. Intercostal block and infiltration with local anaesthetics of the surgical wound were not performed for all patients.

### Psychoeducational intervention

Patients in the control group received general education support, including leaflets and behavioural instructions, regarding appropriate ways they could adhere to medical advice to support their recovery.

In addition to general educational support, PG patients received psychological evaluations and interventions before surgery. First, the psychological symptoms of the PG patients were assessed with the symptom checklist-90 (SCL-90) questionnaire. In the SCL-90 inventory, 90 items are scored on a five-point scale to reflect the psychological symptom patterns of the patient. The items refer to the assessment of 10 indices, one each for somatization, obsessive-compulsive tendencies, depression, anxiety, phobia, interpersonal sensitivity, hostility, paranoid ideations, psychotic states, and other symptoms. Patients with any index scored above 1 were identified as having psychological disorders, and individualized psychological interventions were administered accordingly. Interventions included encouragement, verbal suggestion, relaxation training, guided imagery and audio or video recordings according to the patient’s individual situation. To ensure the outcome of the interventions, the procedure was performed by two trained nurses with 5 and 7 years of professional experience.

### Outcome variables

The following data were collected for this study: acute postoperative pain scores during the first 3 PODs, opioid consumption for acute pain after surgery, the incidence of rescue analgesia, side effects of the opioid medications, and Grade 2 or higher postoperative complications.

A numeric rating scale (NRS; 0, no pain; 10, worst pain imaginable) was used to evaluate acute pain after surgery, which was measured at 2, 24, 48, and 72 h after surgery by registered nurses. The amount of opioid medication used for acute pain during the first 3 PODs, the incidence of rescue analgesia, and opioid-related side effects were collected by the anaesthesiologists. Opioid consumption was converted to morphine equivalents (mg) using a standard conversion ratio. Postoperative complications were assessed and recorded by the surgeons using the Clavien–Dindo grading system, in which Grade 2 or higher complications were defined as requiring pharmacological or surgical treatment and/or presentation with life-threatening complications or death.

### Statistical analysis

The Pearson chi-squared test was used to compare categorical variables. Differences in continuous variables between the PG and CG groups were assessed using Student’s t test. All statistical analyses were performed using SPSS software, version 2.0.0 (IBM Corporation, Armonk, NY, USA). A *P* value < 0.05 was accepted as statistically significant.

## Results

### Psychological disorders of PG patients

The psychological disorders of PG patients measured with SCL-90 (scored above 1) before surgery are listed in Table 2. The most common psychological disorders were anxiety and interpersonal sensitivity, which were recorded from 82.9% (63/76) and 63.2% (48/76) of patients, respectively. In addition, hostility and obsessive-compulsive tendencies occurred in 10 (13.2%) and 6 (7.9%) patients, respectively.

**Table 2:** Psychological disorders of PG patients measured with SCL-90 before surgery

Indexes	Number of patients scored above 1, n(%)
Somatization	0(0)
Obsessive-compulsive tendencies	6(7.9)
Depression	4(5.3)
Anxiety	63(82.9)
Phobia	2(2.6)
Interpersonal sensitivity	48(63.2)
Hostility	10(13.2)
Paranoid ideations	5(6.6)
Psychotic states	0(0)
Other	3(3.9)

*Abbreviations*: *PG* psychoeducational intervention group, *SCL-90* symptom checklist-90

### Acute pain scores after surgery

**Fig. 1:**
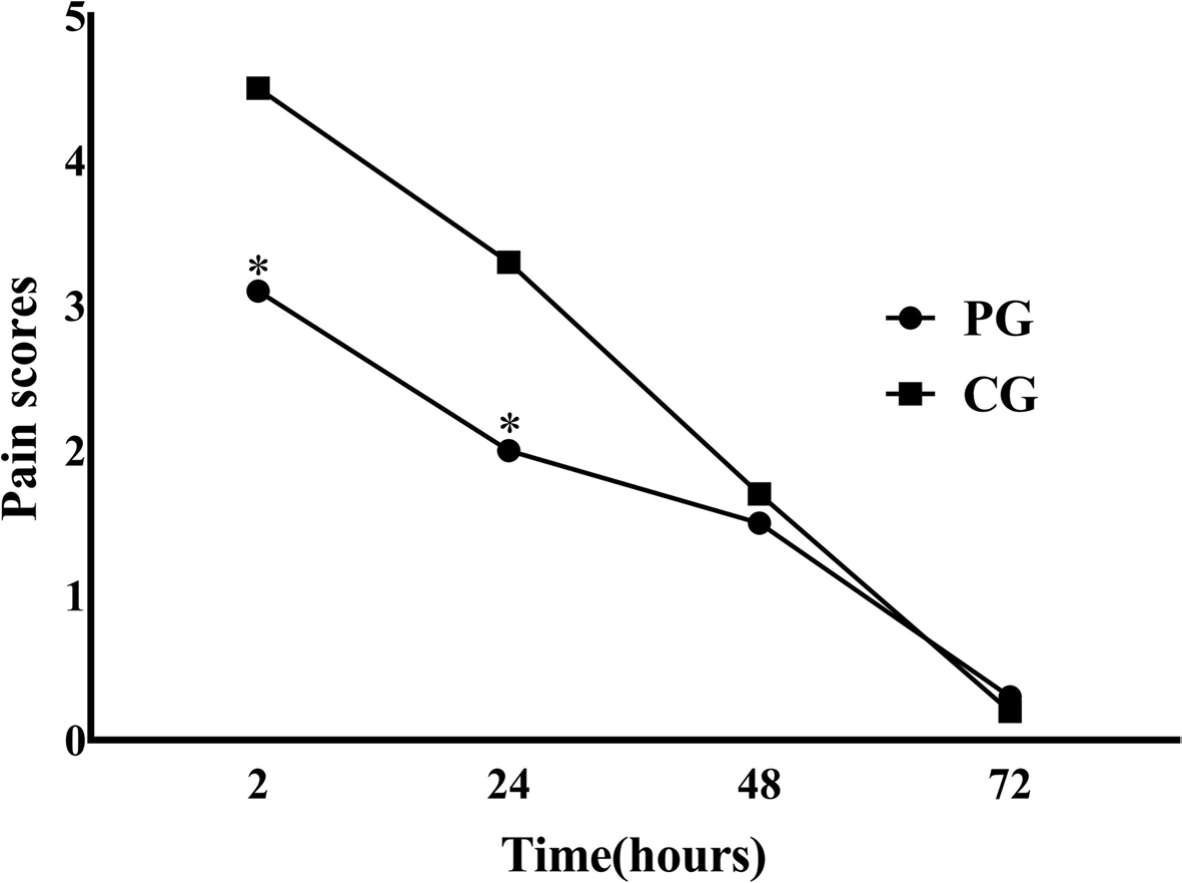
NRS pain scores during the first 3 PODs in both groups. *P < .001, vs. CG group at indicated time points after surgery. NRS, numeric rating scale; POD, postoperative day; PG, psychoeducational intervention group; CG, control group

Table 3 and Fig. 1 illustrate the comparison of acute pain scores during the first 3 PODs in both groups. The results showed that the worst pain was experienced at 2 h after surgery, with the severity alleviating over time. We also found that the NRS pain scores in the PG were significantly lower than those in the CG at 2 and 24 h after surgery (*P* < 0.001).

**Table 3:** NRS pain scores during the first 3 PODs in both groups

Variable	Score, mean ± SD		***P***-value
	PG	CG	
2 h	3.1 ± 1.2	4.5 ± 1.3	< 0.001
24 h	2.0 ± 0.5	3.3 ± 0.7	< 0.001
48 h	1.5 ± 0.4	1.7 ± 1.0	0.106
72 h	0.3 ± 0.1	0.2 ± 0.1	0.155

*Abbreviations*: *NRS* numeric rating scale, *POD* postoperative day, *SD* standard deviation, *PG* psychoeducational intervention group, *CG* control group

### Opioid consumption for acute postoperative pain and the incidence of rescue analgesia

As detailed in Table 4, the total opioid consumption for acute pain after surgery in the PG was 52.1 mg of morphine equivalent, which was significantly lower than that (67.8 mg) in the CG (*P* = 0.038). The results also demonstrated that PG patients had a significantly lower incidence of rescue analgesia than CG patients (28.9% vs. 44.7%, *P* = 0.044).

**Table 4:** Total opioid consumption for acute pain after surgery and the incidence of rescue analgesia

Variable	PG	CG	***P***-value
Total opioid consumption (morphine equivalent, mg)^a^	52.1 ± 15.5	67.8 ± 20.9	0.038
Rescue analgesia, n(%)	22(28.9)	34(44.7)	0.044

*Abbreviations*: *PG* psychoeducational intervention group, *CG* control group

^a^Data presented as mean ± standard deviation (SD)

### Side effects of the opioid medications

The opioid-related side effects recorded in the two groups are listed in Table 5. Altogether, nausea/vomiting was the most common adverse event, which was recorded from 3 (3.9%) PG patients and 10 (13.2%) CG patients (*P* = 0.042). In addition, dizziness was observed in 4 (5.3%) patients in the intervention group and 5 (6.6%) patients in the group that did not receive psychoeducational intervention, and the difference was not statistically significant (*P* = 0.731). We also found that the incorporation of psychoeducational intervention in our cohort showed significantly reduced total opioid-related adverse events compared with the CG (11.8% vs. 25.0%, *P* = 0.036).

**Table 5:** Side effects of the opioid medications

Side effects	Number of patients, n(%)		***P***-value
	PG	CG	
Nausea/vomiting	3(3.9)	10(13.2)	0.042
Dizziness	4(5.3)	5(6.6)	0.731
Hypersomnia	1(1.3)	2(2.6)	0.560
Headache	1(1.3)	1(1.3)	1.000
Respiratory depression	0(0)	1(1.3)	–
Urinary retention	0(0)	0(0)	–
Pruritus	0(0)	0(0)	–
Total	9(11.8)	19(25.0)	0.036

*Abbreviations*: *PG* psychoeducational intervention group, *CG* control group

### Postoperative complications

As shown in Table 6, no significant difference was observed between PG and CG patients in terms of grade 2 or higher postoperative complications (10.5% vs. 17.1%, *P* = 0.240), although there was a trend of reduction for atelectasis, recorded in 1 (1.3%) PG patient and 4 (5.3%) CG patients (*P* = 0.172).

**Table 6:** Grade 2 or higher postoperative complications

Complications	Number of patients, n(%)		***P***-value
	PG	CG	
Atelectasis	1(1.3)	4(5.3)	0.172
Pneumothorax	2(2.6)	3(3.9)	0.649
Pleural effusion	2(2.6)	2(2.6)	1.000
Arrhythmia	2(2.6)	1(1.3)	0.560
Hemorrhages	1(1.3)	2(2.6)	0.560
Pulmonary infection	0(0)	1(1.3)	–
Chylothorax	0(0)	0(0)	–
Pulmonary embolism	0(0)	0(0)	–
Total	8(10.5)	13(17.1)	0.240

*Abbreviations*: *PG* psychoeducational intervention group, *CG* control group

## Discussion

Postoperative pain management is an important component of patient care. In this study, the combined analgesia modality of IV-PCA plus psychoeducational intervention was retrospectively evaluated for patients with pulmonary nodules treated by thoracoscopic surgery. The results showed that the combined analgesia resulted in reduced acute postoperative pain, less opioid consumption and fewer opioid-related side effects than IV-PCA alone.

The perception and severity of postoperative pain are influenced by various biological and psychosocial factors [15, 16]. It has been reported that patients with perioperative anxiety and depression demonstrate more serious acute pain after surgery and larger analgesic requirements [11, 17, 18]. A positive and confident attitude towards surgery and anaesthesia is associated with reduced anxiety and improved postoperative behavioural activation and thus might alleviate pain [19]. Consequently, the incorporation of psychoeducational intervention into a standard analgesia protocol seems to be an attractive approach for the management of acute postoperative pain. In our study, up to 82.9 and 63.2% of PG patients experienced psychological disorders of anxiety and interpersonal sensitivity, respectively, and as expected, with the addition of psychoeducational intervention, significantly reduced NRS pain scores at 2 and 24 h after surgery were observed with respect to CG patients. In fact, a pain relief effect conferred by psychological intervention has also been observed in patients undergoing open heart surgery [18].

Opioids are the most commonly used analgesics during the postoperative period [6, 20]. However, opioid receptors are distributed throughout the central and peripheral nervous systems, which means that opioid administration can induce effects beyond analgesia. The use of opioids even with PCA is associated with dose-dependent side effects, which limit their effectiveness in some patients [21]. To our excitement, in the current study, PG patients displayed less opioid consumption (morphine equivalent of 52.1 mg vs. 67.8 mg, *P* = 0.038) and fewer opioid-related side effects (11.8% vs. 25.0%, *P* = 0.036) than CG patients. These results, together with the pain relief data mentioned above, further indicate that IV-PCA plus psychoeducational intervention might be a promising method for the management of acute postoperative pain in patients with PN after thoracoscopic surgery.

Notably, although the relationship between postoperative complications and pain intensity has been reported in several studies [8, 22, 23], in our cohort, psychoeducational intervention did not reduce postsurgical complications (*P* = 0.240). Given the relatively small sample size and the selection bias in the patient population, further prospective studies are needed to verify these results.

This study has some limitations. The main limitation arose from the retrospective nature of the data, which were obtained through past records. Another drawback was the potential bias introduced by patient selection. Furthermore, the psychological symptoms of PG patients were not reassessed after psychoeducational intervention, so the outcomes of the intervention, whether it was truly beneficial or not, could not be analysed in the present study. Additionally, only intravenous analgesia was used, involving administration of three different opioids (albeit equipotent). Finally, observation bias, also known as the Hawthorne effect, which concerns research participation, the consequent awareness of being studied, and possible impact on behaviour, is a disadvantage of this study.

## Conclusions

Psychoeducational intervention for PN patients treated by thoracoscopic surgery resulted in reduced acute postoperative pain, less opioid consumption and fewer opioid-related side effects. Further clinical trials are needed to confirm these findings.

## Data Availability

The datasets used or/and analysed during the current study are available from the corresponding author on reasonable request.

## Abbreviations

PN: Pulmonary nodule
VATS: Video-assisted thoracoscopic surgery
IV-PCA: Intravenous patient-controlled analgesia
PG: Psychoeducational intervention group
CG: Control group
ASA-PS: American Society of Anesthesiologists physical status
POD: Postoperative day
